# LGI‐1 Encephalitis: A Comprehensive Multidisciplinary Paradigm for Diagnosis and Management

**DOI:** 10.1002/alz.089908

**Published:** 2025-01-09

**Authors:** Emytis Tavakoli, Keera Fishman, Sarah Elmi

**Affiliations:** ^1^ CAMH, Toronto, ON Canada; ^2^ Baycrest, Toronto, ON Canada; ^3^ Ontario Shores Centre for Mental Health Sciences, Whitby, ON Canada

**Keywords:** autoimmune limbic encephalitis, cognitive impairment, hyponatremia, rituximab, LGI‐1 antibody encephalitis, multidisciplinary treatment

## Abstract

**Introduction:**

Leucine‐rich glioma‐inactivated 1 (LGI‐1) antibody encephalitis is a rare subtype of autoimmune limb encephalitis (ALE), which is marked by rapid neuropsychiatric decline. This report details a comprehensive approach to its diagnosis and management.

**Assessment:**

In this case, a 68‐year‐old man presented with aggressive behaviors, cognitive decline, and seizure‐like episodes. Initial assessments suggested major neurocognitive disorder, with MMSE and MOCA scores of 20 and 17, respectively, indicating cognitive impairment. EEG confirmed episodes of facio‐brachial dystonic seizures. Further evaluation revealed hyponatremia, temporal lobe atrophy on MRI, and LGI1‐antibodies in the CSF.

**Treatment:**

Initial treatments included steroids and IVIG, which did not result in significant improvement. However, after receiving two Rituximab trials, improvements in cognitive and behavioural outcomes were observed. Cognitive predictors of poor outcomes included older age, non‐response to initial therapies, and clinical relapses.

**Discussion:**

This case underscores the complex presentation of LGI‐1 encephalitis, emphasizing the importance of thorough diagnostic evaluations and a multimodal treatment approach. The patient’s cognitive and behavioral improvements post‐treatment highlight the significance of timely and targeted interventions in managing this challenging autoimmune encephalitis. Long‐term follow‐up revealed sustained improvements, reinforcing the potential efficacy of a multidisciplinary treatment strategy.

